# The Prevalence of Hemoglobinopathies in Reference Laboratory of Kermanshah, Western Iran

**Published:** 2019-02

**Authors:** Zohreh RAHIMI, Sozan NAJAFI, Lila MOGHOFEHIE, Ebrahim AMIRI, Asad VAISI-RAYGANI, Ziba RAHIMI

**Affiliations:** 1. Medical Biology Research Center, Kermanshah University of Medical Sciences, Kermanshah, Iran; 2. Department of Clinical Biochemistry, Faculty of Medicine, Kermanshah University of Medical Sciences, Kermanshah, Iran; 3. Student Research Committee, Faculty of Medicine, Kermanshah University of Medical Sciences, Kermanshah, Iran; 4. Central Lab, Kermanshah University of Medical Sciences, Kermanshah, Iran

## Dear Editor-in-Chief

Hemoglobinopathies are a group of autosomal recessive disorders that consisted of structural variants of hemoglobins (S, C, D, O, Q, Setif) and thalassemia syndromes (*α*- and *β*-thalassemia) (1). The hemoglobin S (Hb S) is the most clinically important structural variant of hemoglobin that results from substitution of va-line for glutamic acid at position 6 of the *β*-chain (2). The Hb D-Punjab (D-Los Angeles) results from substitution of glutamine for glutamic acid at position of 121 of the *β*-globin chain (3). Hb Q disorders are structural variants of α-chain including Hb Q-Iran, Hb Q-Thailand, and Hb Q-India. Hb Setif is another α-chain hemoglobin variant with electrophoretic mobility similar to Hb S at alkaline pH (4). Thalassaemias are heterogeneous disorders characterized by decrease or the absence of production of *α*-(*α*- thalassemia) or *β*-globin chains (*β*-thalassemia) (1). Beta thalassemia is the most common genetic disorder in Iran with two millions carriers in the country (5). In individuals who referred to Gholhak Clinical Laboratory (Tehran, Iran) the prevalence of minor β-thalassemia was around 20.7% (6).

During Apr 2016 to Mar 2017, 4065 individuals including 2179 females (mean age 26.2±17.3 yr) and 1886 males (mean age 26.3±19.8 yr) referred to the Reference Laboratory of the Kermanshah University of Medical Sciences. From the file of individuals’ sex, age and hematological indices were extracted. Hemoglobin fractions were separated in silica capillaries using Sebia capillary zone electrophoresis. The SPSS software ver. 22 (Chicago, IL, USA) was used for the statistical analysis.

Overall 1053 subjects (25.9%) were with anemia (Hb<12 g/dl). Among men 326 out of 1886 (17.3%) and in women 727 out of 2179 (33.4%) had anemia. Abnormal hemoglobin fractions were detected in 876 out of 4065 (21.5%) individuals. The minor β-thalassemia was found in 757 (18.6%) subjects as the most prevalent hemoglobinopathy and was detected in 324 males and 433 females with the mean Hb A_2_ levels of 5.5±0.64% and 5.4%±0.56%, respectively. The most prevalent Hb variants were Hb D (0.96%), Hb Q (0.49%), and Hb Setif (0.37%) (Table 1). There were 39 individuals with Hb D including 18 males and 21 females. Thirty-five out of 39 were carrier of Hb D with the mean levels of 40.4%±2.2% and 38.2%±2.1% in males and females, respectively. One homozygote Hb D with normal hematological indices and Hb D level of 95.1% was detected. The remaining 3 individuals were compound heterozygote for HbD/β-thalassemia with a picture of minor beta thalassemia. Hb S was detected in one individual (0.025%) with the mean level of 44.8%. Hb Q was found in 20 individuals (13 males and 7 females) with the mean percentage of 19.5±0.6% in males and 19.1±0.32% in females. Hb Setif was detected in 10 males with the mean of 16±1.1% and in 5 females with the mean of 15.9±0.65%. Three individuals consisted of a male and 2 females were with Hb Arya with the level of 19 and 12.2%±0.56%, respectively. Hb H was found in 3 males and 9 females with the levels of 4.96±3.6 and 7.5±5.8%. Hb Barts was observed in 3 males (0.83±0.58%) and 6 females (1.31±1.5%). The Hb Constant Spring was found in 2 females with the mean level of 0.85±0.21%. The mean Hb J (α-chain variant) level in 2 males was 39.4±17.3 % and in two females was 39.4±15.9%. Hb X was detected in 6 males (27.07±15.6%) and 7 females (18.9±12.8%) (Table 1).

**Table 1:** The frequency of abnormal hemoglobins in studied individuals

**Hemoglobin variants**	**N**	**%**
The minor β-thalassemia	757	18.6
Hb D disease	39	0.96
Hb Q trait	20	0.49
Hb Setif trait	15	0.37
Hb S trait	1	0.025
Hb J trait	4	0.1
Hb Constant Spring	2	0.05
Hb H disease	12	0.3
Hb Barts	9	0.22
Hb Arya	3	0.07
Hb X	3	0.07
X	11	0.27

Previously, Hb D-Punjab was the most prevalent β-globin chain structural variant in the Kermanshah Province followed in frequency by α-chain variants of Hb Q-Iran and Hb Setif (7). Similarly, in the present study, the common structural variant was Hb D (0.96%) followed by Hb Q (0.49%) and Hb Setif (0.37%). Although, we could not detect the type of Hb D and Hb Q but according to our previous report (7) it seems these hemoglobin variants be Hb D-Punjab and Hb Q-Iran.

We found a high frequency of β-thalassemia trait (18.6%) that indicates the screening program in the country and Kermanshah province needs to be continuing for this hemoglobinopathy. The second hemoglobinopathy in our area was Hb D.

## References

[1] Rahimi Z (2013). Genetic epidemiology, hemato-logical and clinical features of hemoglobinopathies in Iran. Biomed Res Int, 2013:803487. 23853772 10.1155/2013/803487 PMC3703361

[2] Rahimi Z Merat A Gerard N (2006). Implications of the genetic epidemiology of globin haplotypes linked to the sickle cell gene in southern Iran. Hum Biol, 78(6):719–31. 17564250 10.1353/hub.2007.0016

[3] Rahimi Z Akramipour R Nagel RL (2006). The beta-globin gene haplotypes associated with Hb D-Los Angeles [beta121 (GH4) Glu-->Gln] in Western Iran. Hemoglobin, 30(1):39–44. 16540414 10.1080/03630260500454105

[4] Rahimi Z Rezaei M L Nagel R Muniz A (2008). Molecular and hematologic analysis of hemoglobin Q-Iran and hemoglobin Setif in Iranian families. Arch Iran Med, 11(4):382–6. 18588369

[5] Rahimi Z Muniz A Parsian A (2010). Detection of responsible mutations for beta thalassemia in the Kermanshah Province of Iran using PCR-based techniques. Mol Biol Rep, 37(1):149–54. 19437135 10.1007/s11033-009-9560-0

[6] Hosseini S Kalantar E Dorgalaleh A (2014). A long term screening of Iranian populations with thalassemia and hemoglobinopathies. Br Med Bull, 2(4): 669–676.

[7] Rahimi Z Muniz A Mozafari H (2010). Abnormal hemoglobins among Kurdish population of Western Iran: hematological and molecular features. Mol Biol Rep, 37(1):51–57. 19333781 10.1007/s11033-009-9516-4

